# Harnessing the Potential of CRISPR/Cas in Targeted Alfalfa Improvement for Stress Resilience

**DOI:** 10.3390/ijms26073311

**Published:** 2025-04-02

**Authors:** Shugao Fan, Linyan Jia, Jiawei Wu, Ying Zhao

**Affiliations:** 1 School of Hydraulic and Civil Engineering, Ludong University, Yantai 264025, China; 3455@ldu.edu.cn (S.F.); wjw2097046621@163.com (J.W.); 2 School of Resources and Environmental Engineering, Ludong University, Yantai 264025, China; j1251546376@163.com

**Keywords:** climate change, genome editing, abiotic stress, plant breeding

## Abstract

Alfalfa (*Medicago sativa*), recognized as the most valuable legume feed crop, faces significant challenges in enhancing both qualitative and quantitative production amidst the pressures of climate change. This review highlights these challenges, including the underutilization of genomic and genetic resources, while proposing potential solutions through genome editing. Our focus is on leveraging CRISPR/Cas technology in conjunction with decades of advancements in conventional breeding to expedite the improvement of alfalfa. By adopting this approach, we aim to overcome the limitations of traditional alfalfa improvement approaches and accelerate the development of improved cultivars capable of thriving in changing climates. Key candidate traits for CRISPR/Cas genome editing, as reviewed in the latest literature, include nutrient use efficiency, freezing tolerance, and resistance to pests and diseases. We dissect literature on potential gene pathways associated with these traits, providing molecular breeders with valuable insights for utilizing CRISPR/Cas genome editing. Furthermore, we propose editing modalities to expedite the development of stress-resilient, genome-edited alfalfa that can effectively cope with climate change.

## 1. Introduction

Alfalfa is a perennial legume with promising economic prospects due to its versatile applications across multiple industries [1]. Primarily valued as a high-quality forage crop, alfalfa serves as a critical feed source for various livestock, contributing significantly to the profitability of the animal agriculture sector [2]. Its ability to fix nitrogen enriches soil fertility, reducing dependency on costly synthetic fertilizers and enhancing overall agricultural sustainability [3]. Additionally, alfalfa’s adaptability extends to biofuel production, erosion control, and soil remediation efforts, amplifying its economic significance beyond traditional agricultural practices [4]. With increasing demand for nutritious animal feed and sustainable farming solutions, alfalfa remains a lucrative crop with ample opportunities for growth and innovation.

Like most crops, sustainable alfalfa improvement must be addressed from a climate change viewpoint with adoption of more effective technology. Genome editing holds significant potential to revolutionize alfalfa breeding by offering precise and targeted genetic modifications to improve desirable traits and address various challenges [5]. CRISPR genome editing accelerates the breeding process by enabling rapid, precise, and predictable genetic modifications without the need for traditional breeding methods. For example, molecular breeders can use CRISPR to enhance traits such as yield, quality, stress tolerance, pest resistance, and nutrient efficiency, leading to the development of improved alfalfa cultivars. Technically breeders can introduce new functionalities, such as enhanced disease resistance, improved nutritional quality, or altered growth habits, by precisely engineering the alfalfa genome using CRISPR technology. Through a knockout approach CRISPR can be used to reduce or eliminate antiquality factors in alfalfa, such as lignin content or alkaloid levels, which can negatively impact forage digestibility and livestock performance [6].

## 2. Leveraging Genomic Resources for Alfalfa CRISPR Editing

Alfalfa exhibits significant intra-species genetic diversity [7]. This genetic heterogeneity complicates the breeding of polygenic traits like biomass yield, persistence, and disease resistance, leading to variability in trait expression and unpredictable outcomes in breeding crosses [8]. Conventional crop improvement strategies often struggle with this complexity, limiting predictable trait improvements. Advances in high-throughput sequencing, such as next-generation and long-read sequencing, have produced valuable genomic resources for alfalfa, including high-quality reference genomes, transcriptomic profiles, and detailed genetic maps [9]. These datasets provide crucial insights into the structure, organization, and function of the alfalfa genome, aiding in the identification of genes associated with important agronomic traits. CRISPR-mediated editing can exploit these resources by enabling the precise modification of key regulatory and coding regions, enhancing the efficiency of trait introgression and gene pyramiding in breeding programs. QTL mapping and genome-wide association studies (GWASs) have played critical roles in dissecting the genetic architecture of traits such as forage yield, persistence, and tolerance to biotic and abiotic stresses [10,11,12]. By combining CRISPR genome editing with QTL and GWAS data, we can validate candidate genes, introduce beneficial alleles, and fine-tune the expression of genes controlling key agronomic traits. This strategy overcomes the limitations of traditional marker-assisted selection (MAS), allowing for precise gene editing and faster phenotypic improvements [13]. Despite the availability of draft genome sequences, gaps in alfalfa’s genome assembly and incomplete functional annotation remain significant obstacles to fully understanding the molecular mechanisms underlying important traits [14].

**Figure 1:**
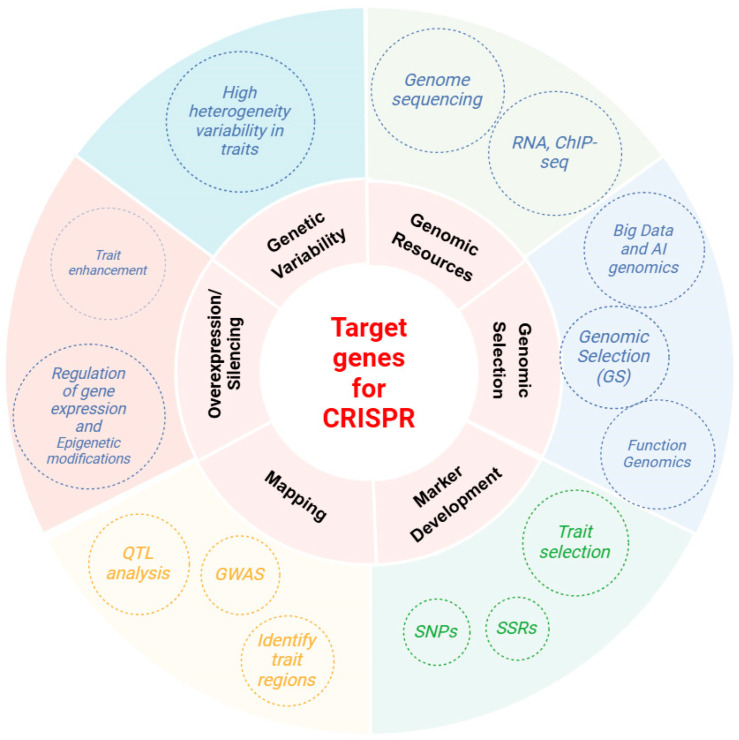
Overview of genomic pathways for alfalfa trait enhancement: integrating gene overexpression, genetic variability, genomic resources, and marker development to identify target genes for CRISPR.

Further, integrating CRISPR genome editing with omics-based approaches, such as transcriptomics, proteomics, and metabolomics, will accelerate the identification of key metabolic pathways and regulatory networks controlling complex traits in alfalfa. This integrative approach will enable the fine-mapping of genotype–phenotype associations, the elucidation of gene-to-trait relationships, and the prioritization of candidate genes for breeding targets. The application of CRISPR-driven functional validation will not only enhance our understanding of the alfalfa genome but also aid in refining genome assemblies and improving the annotation of non-coding regulatory elements (Figure 1).

## 3. Examples of CRISPR Target Traits and Editing Modalities

### 3.1. Knockout of Biotic Stress Tolerance Genes

Alfalfa is susceptible to a wide range of diseases and pests, which can significantly reduce yield and forage quality, leading to substantial economic losses for growers. Common pathogens include alfalfa mosaic virus, root rot, anthracnose, bacterial wilt, and various leaf spot diseases, while pests such as aphids, leafhoppers, and alfalfa weevils exacerbate crop damage [15,16]. Climate variability and shifting weather patterns further complicate disease and pest dynamics, making management efforts more unpredictable and challenging [17]. Breeding for resistance or tolerance to these biotic stresses is therefore critical for developing resilient alfalfa cultivars. However, this goal is challenging due to the complex host–pathogen interactions and the diversity of pathogens affecting alfalfa [18]. The application of CRISPR/Cas genome editing allows researchers to directly manipulate specific genes implicated in host defense mechanisms, thus bypassing the limitations of conventional breeding. For example, *AtEFR*, which confers resistance to bacterial wilt, and the lactoferrin gene, associated with resistance to bacterial stem blight, represent promising targets for CRISPR-mediated enhancement of disease resistance [19,20]. QTL mapping and gene discovery efforts have uncovered genes linked to resistance against *Phytophthora* root rot and *Aphanomyces* root rot, two of the most damaging root diseases in alfalfa [21,22]. These genes, particularly those with broad-spectrum resistance, are ideal candidates for CRISPR/Cas editing to develop cultivars with enhanced resistance. Functional genomics studies, such as the overexpression of *MtRAM2* resulting in increased susceptibility to *Phytophthora* root rot, provide further insights into potential susceptibility genes that can be targeted for knockout using CRISPR to mitigate vulnerability [23]. Research into the genetic basis of resistance to iron deficiency chlorosis and other abiotic stresses is also ongoing, with genes such as those identified by Zamboni [24] being potential targets for genome editing.

### 3.2. Multiplexing Towards Abiotic Stress Tolerance

Achieving abiotic stress tolerance in alfalfa while maintaining high yield and forage quality is a significant challenge due to the broad range of environmental conditions under which it is cultivated [25]. Traits such as stress tolerance, yield stability, and forage quality are all influenced by the interaction between genotype, environment, and management practices [26]. To ensure stable productivity, alfalfa must perform consistently across diverse climates and soils, making it essential to breed for broad adaptability. The challenge is to breed cultivars that can withstand abiotic stress while maintaining the high nutritional standards required by livestock producers [27]. The real potential of CRISPR lies in its ability to perform multiplex genome editing. Multiplex gene editing enables the simultaneous modification of multiple loci [28]. This is particularly valuable for polygenic traits like disease resistance, where multiple genes and pathways often contribute to the phenotype. One of the major challenges in breeding alfalfa for abiotic stress tolerance lies in the need to balance multiple traits simultaneously.

Multiplexing allows for the simultaneous modification of multiple genes, which is essential for addressing the polygenic nature of abiotic stress tolerance. Many abiotic stress responses in plants are governed by complex gene networks that regulate processes such as osmotic balance, stress signaling, and antioxidant defense. These networks involve numerous genes, each playing a role in the plant’s ability to tolerate various stresses. With multiplex CRISPR editing, breeders can target multiple key genes across these networks in a single step, thereby accelerating the development of stress-tolerant cultivars.

Drought tolerance, for example, is a highly complex trait influenced by several physiological mechanisms, including stomatal regulation, water use efficiency, and root architecture. These processes are influenced by signaling pathways such as the MAPK and Ca^2+^ signaling pathways, the ABA-mediated signaling pathway, and key transcription factors (TFs) like MYB, WRKY, ERF, NAC, and bZIP [29]. Similarly, cold tolerance in alfalfa involves genetic pathways that regulate the plant’s ability to mitigate oxidative stress and prevent cellular damage during freezing temperatures. This includes the Ca^2+^ signaling pathway and the cold-regulated (COR), inducer of CBF expression (ICE), and C-repeat binding factor (CBF) genes, which play crucial roles in cold acclimation and freezing tolerance. Recent research has identified genes linked to freezing tolerance, such as those involved in the reactive oxygen species (ROS) pathway and osmotic regulation [30]. Using multiplex CRISPR editing, these genes can be simultaneously modified to enhance cold hardiness without compromising other agronomic traits such as yield potential. Additionally, proteomic studies have identified proteins involved in heat stress adaptation, presenting further targets for multiplex editing to improve heat tolerance in alfalfa [31].

Another significant advantage of multiplex genome editing is the ability to improve broad-spectrum abiotic stress tolerance [32]. By simultaneously targeting key genes across different stress response pathways, breeders can develop cultivars with improved resistance to multiple stresses, including drought (DREB, AREB, PP2C, SnRK2), heat (Hsf, HSPs), cold (CBF, COR, ICE, LOS), and salinity (NHX, SOS1, HKT, AVP1). This broad-spectrum tolerance is essential for crops like alfalfa, which are cultivated across regions with highly variable climatic conditions. For example, germplasm evaluations have identified sources of drought tolerance, salinity resistance, and cold hardiness in wild relatives of alfalfa [33].

In addition to its applications in multiplex genome editing, CRISPR can be integrated with other advanced breeding techniques, such as genomic selection and marker-assisted breeding. Genomic selection leverages prediction models that combine genomic data with phenotypic information to estimate genomic estimated breeding values (GEBVs) for traits such as stress tolerance and yield stability [34]. By incorporating CRISPR-mediated edits into these models, breeders can accelerate the selection of individuals with optimal trait combinations, reducing the reliance on extensive field trials. This integration of CRISPR with genomic selection enhances the efficiency of breeding programs by rapidly introgressing stress resistance alleles into elite breeding lines [35]. Moreover, the use of high-throughput phenotyping platforms can further streamline the selection process in alfalfa breeding programs. These platforms enable the rapid assessment of plant performance under various stress conditions, allowing breeders to screen large populations for desirable traits. When combined with CRISPR’s precision, this high-throughput approach enhances the ability to select plants with superior stress tolerance and high yield potential.

CRISPR/Cas genome editing, particularly in its multiplex form, represents a powerful tool for overcoming the challenges associated with breeding alfalfa for abiotic stress tolerance. By enabling precise and simultaneous modifications of multiple genes, CRISPR allows breeders to address the polygenic nature of stress responses, leading to cultivars with improved resilience, yield stability, and forage quality. The integration of multiplex genome editing with advanced breeding technologies such as genomic selection further enhances the efficiency of breeding programs, paving the way for the development of alfalfa cultivars that can thrive in increasingly unpredictable environmental conditions.

### 3.3. Editing for Nutrient Uptake Efficiency

Fertilizer usage efficiency is a critical target trait for any crop improvement program [36]. While alfalfa can fix atmosphere nitrogen to meet its high production demands, optimum germination requires an initial fertilizer supply [37]. Plant nutrition should primarily focus on three critical nutrients: nitrogen (N), phosphorus (P), and potassium (K). Ensuring adequate levels of N, P, and K in the soil is crucial for optimizing crop yield and quality [38]. The nitrogen transporter *NRT1.1* gene serves as an auxin carrier that reduces auxin levels in the lateral root apex to limit lateral root extension in nitrogen-deficient soil [39]. In another study, it was reported that this process occurs upstream of the *ANR1* gene, which initiated a signaling cascade that promoted lateral root elongation towards the high-nitrogen zone in the rhizosphere [40,41]. Nitrogen deficiency in the soil like that caused by salt and drought causes the root to produce C-terminally encoded peptides (CEPs), which are transported to the shoot via the xylem and are recognized by two leucine-rich repeat (LRR) receptor kinases, CEP Receptor 1 and 2 (CEPR1/2) [42]. This causes the upregulation of Downstream1 and 2 (CEPD1/2), which are carried to the roots and foster nitrogen uptake by upregulating the high-affinity N transporter gene *NRT2.1* in nitrogen-deficient tissue [43].

**Figure 2:**
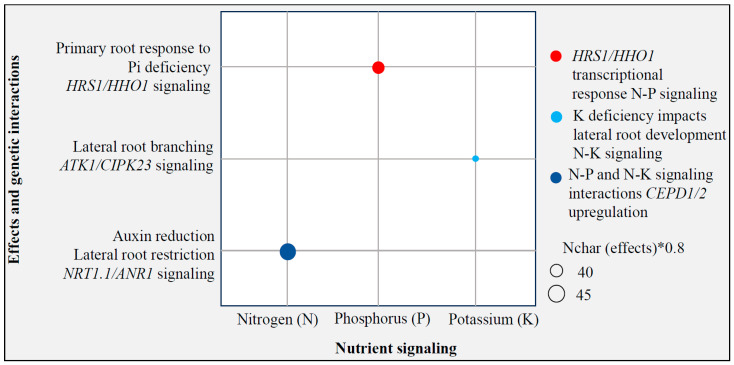
Bubble plot showing transcriptional regulation of N and P uptake to indicate that target genes for fertilizer uptake efficiency lie within the signal perception, transduction, and response mechanism to low N and P.

Surprisingly, there is evidence demonstrating close relationships between nitrogen and phosphate (Pi) signaling to influence plant physiological and developmental processes [44,45]. The *GRAS* family transcription factor gene *HRS1* and its paralog *HHO1* have been shown to be transcriptionally induced by N but post-translationally regulated by Pi deficiency before coordinating a N-dependent primary root response to Pi deficiency [44]. Evidence also suggests that N and K signaling interact. For example, low K inhibits primary root growth, which is dependent on NH_4_^+^ [46]. Another N-K interaction aspect emerges in the control of lateral root branching. It was demonstrated that low nitrogen levels can effectively reduce secondary lateral root development driven by K deficiency [47]. An analysis of root systems in mutants deficient in genes known to be involved in K and N transport and/or signaling reveals that the N transporter *NTR1.1/NPF6.3* and the K channel *AKT1* play critical roles in this root response [47]. Also, *CIPK23* was shown to play a connective role that integrates N and K signals to control lateral root development via phosphorylation of *NTR1.1* and *AKT1* [47]. These observations incorporating N-P-K at the genetic level are important starting points in genome editing toward improved fertilizer uptake efficiency (Figure 2).

In the context of enhancing fertilizer use efficiency through nutrient signaling and uptake, several genes have emerged as promising targets for CRISPR-mediated genome editing. One such target is *NRT1.1* (*NPF6.3*), a nitrogen transporter involved in auxin transport and lateral root development, especially under nitrogen-deficient conditions. Editing this gene could improve nitrogen uptake and optimize root architecture in nitrogen-poor soils, potentially boosting crop performance in low-fertilizer environments. Another key candidate is *NRT2.1*, which plays a vital role in high-affinity nitrogen uptake. Enhancing its expression or modifying its nitrogen affinity could significantly increase nitrogen uptake efficiency, providing plants with better access to this essential nutrient [48].

**Figure 3:**
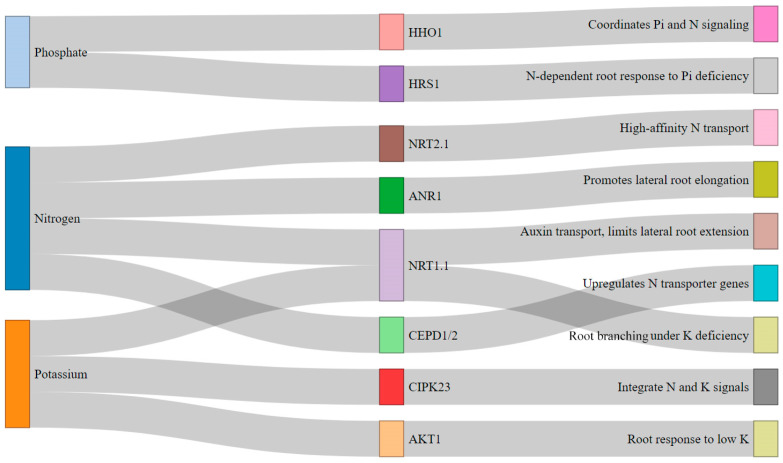
Alluvial plot showing pathways for modulating nitrogen, phosphate, and potassium signaling pathways: Key genes and their expected outcomes in root development and nutrient uptake in *Medicago sativa* (alfalfa). Key genes include Homeodomain leucine zipper protein 1 (*HHO1*), High-affinity phosphate transporter 1 (*HRS1*), Nitrate transporter 2.1 (*NRT2.1*), AUXIN RESPONSE FACTOR 1 (*ANR1*), Nitrate transporter 1 (*NRT1.1* 1), CEP domain-containing proteins 1 and 2 (*CEPD1/2*), CBL-interacting protein kinase 23 (*CIPK23*), and Arabidopsis potassium channel 1 (*AKT1*).

Beyond nitrogen-related genes, the *AKT1* potassium channel is crucial for potassium transport within roots [49]. CRISPR editing of *AKT1* could enhance potassium uptake and strengthen plant resilience to potassium deficiency, ultimately improving overall fertilizer use efficiency. Additionally, the *HRS1* gene, which regulates plant responses to phosphate (Pi) deficiency, is a promising target. Its interaction with nitrogen signaling suggests that editing *HRS1* could optimize plant responses to both nitrogen and phosphorus deficiencies, promoting nutrient uptake and root growth [50]. Finally, the *CIPK23* gene integrates signals from nitrogen and potassium to regulate root development. Modifying *CIPK23* could fine-tune the plant’s nutrient response, leading to increased crop yields and reduced reliance on excessive fertilizer inputs. Collectively, these targeted genetic modifications could pave the way for more efficient and sustainable agricultural practices (Figure 3).

### 3.4. Antinutrient-Free Knockouts

**Figure 4:**
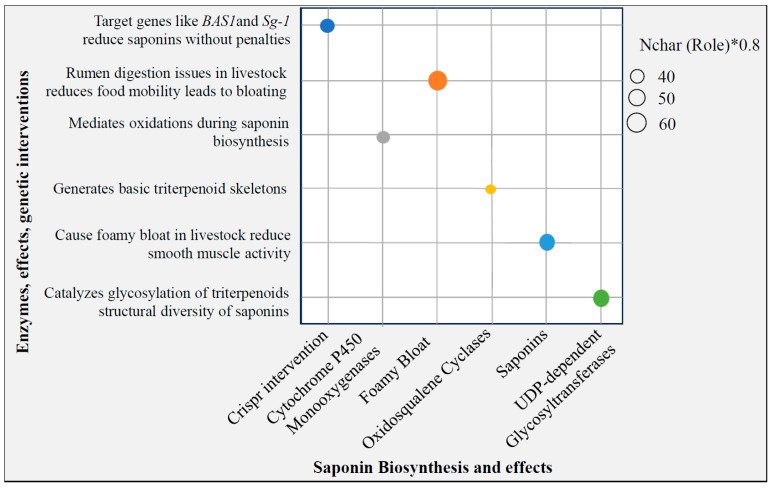
Bubble chart representing the CRISPR and Enzymatic Interventions in Saponin Biosynthesis: Targeting Genes, Enzyme Roles, and Their Effects on Livestock Health and Saponin Diversity. The different bubble colors represent distinct functional categories related to saponin biosynthesis and its effects.

Antinutrients are naturally occurring compounds found in plants that can interfere with nutrient absorption or digestion, potentially causing negative health effects. Alfalfa has antinutrients that cause foamy bloating in livestock [8]. Foamy bloating with alfalfa was associated with a high amount of saponin when ingested by both humans and livestock [51]. Together with other soluble proteins, when saponins are released into the stomach, they reduce smooth muscle activity, thereby reducing food mobility. This reduces the rumen digesta passage rate and may directly reduce or stop the eructation reflex [52]. Saponin biosynthesis requires three critical enzymes: oxidosqualene cyclases, which generate the basic triterpenoid skeletons; cytochrome P450 monooxygenases, which mediate oxidations; and uridine-diphosphate-dependent glycosyltransferases, which catalyze glycosylations [53]. In *Platycodon grandifloras*, the cytochrome P450 monooxygenase CYP716A141 was implicated in the biosynthesis of triterpenoid saponins [54]. Manipulation of saponin biosynthesis using RNAi revealed that β-amyrin synthase gene *BAS1* was positively involved in saponin biosynthesis in soybean as silenced lines were almost devoid of saponins without any other phenotypic penalties. Through a GWAS, the Sg-1 glycosyltransferase locus was found to regulate structural diversity of triterpenoid saponins of soybean [55]. Also, the biosynthesis of DDMP saponins in soybean is regulated by a distinct UDP-glycosyltransferase [56]. These findings pin-point key CRISPR KO to eliminate or reduce saponins without phenotypic penalties (Figure 4). Also, CRISPR-mediated genome editing enables the introduction of genetic changes that enhance alfalfa’s resistance to diseases.

## 4. Technical Bottlenecks and Advances in CRISPR/CAS Application in Alfalfa

Alfalfa (*Medicago sativa*) is characterized by a large and complex genome, presenting significant challenges for CRISPR-mediated genome editing [57]. Its polyploid nature, featuring multiple copies of each chromosome, complicates the precise targeting and editing of specific gene copies, resulting in potential off-target effects and unintended mutations [58]. The efficiency of CRISPR-mediated genome editing in alfalfa can vary based on the delivery method and the efficacy of introducing editing components into plant cells [59]. Conventional delivery methods, such as Agrobacterium-mediated transformation and particle bombardment, may face limitations in efficiency and scalability, particularly in large-scale editing endeavors [60]. Many agronomically important traits in alfalfa—including yield, forage quality, and stress tolerance—are governed by complex interactions among multiple genes [61]. The editing of these traits via CRISPR may necessitate the concurrent targeting of multiple genes or the modification of regulatory elements, which presents both technical and logistical hurdles. Alfalfa transformation typically relies on tissue culture techniques for the regeneration of whole plants from transformed cells. However, the recalcitrance of alfalfa tissue culture often results in the unavailability of efficient regeneration protocols for all genotypes or species, thus constraining the applicability of CRISPR across diverse genetic backgrounds [62].

**Figure 5:**
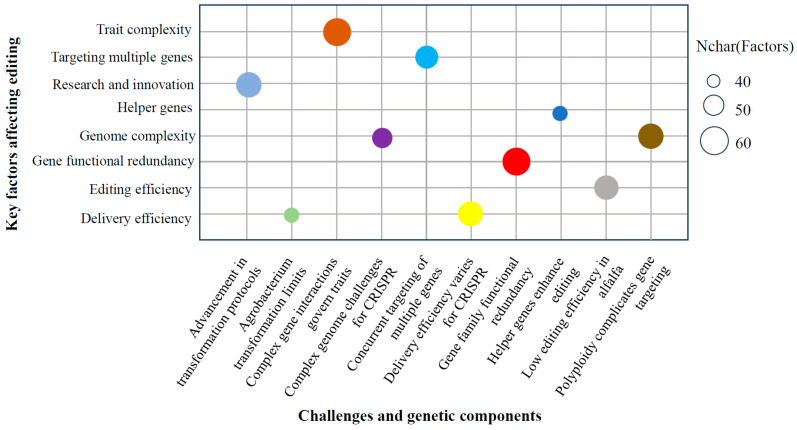
Bubble plot showing bottlenecks and advances in CRISPR genome editing in alfalfa. Different bubble colors represent various categories of challenges and genetic components influencing CRISPR efficiency, such as genome complexity, gene functional redundancy, and editing or delivery efficiency.

Additionally, alfalfa genomes frequently harbor gene families with functional redundancy, wherein multiple genes can perform analogous functions [63]. Targeting a single gene within such a redundant family may not produce the desired phenotype due to compensatory mechanisms involving other family members. Consequently, the editing of multiple genes simultaneously may be essential to achieve specific trait modifications [64]. Addressing these challenges will require ongoing research and innovation in CRISPR technology, alongside advancements in alfalfa transformation protocols, genome sequencing, and bioinformatics tools (Figure 5). Current methodologies have incorporated helper genes, such as Wuschel2, and ultraistic plasmids to enhance editing efficiency. The authors of [65,66] detail comprehensive procedures for generating a non-homologous end-joining-derived indel at a specific genomic locus in alfalfa utilizing CRISPR/Cas9. This method includes critical steps such as guide RNA design, binary CRISPR vector construction, Agrobacterium-mediated transformation of alfalfa explants, and molecular assessment of transformed genotypes for transgene integration and editing confirmation.

Despite these challenges, several breakthroughs have been achieved in alfalfa genome editing. Gao [67] successfully applied the CRISPR/Cas9 technique to induce mutations in the squamosa promoter-binding protein-like 9 (SPL9) gene in alfalfa. Due to the genome’s complexity, they initially employed droplet digital PCR (ddPCR) for high-throughput screening of large populations of CRISPR-modified plants. Based on the genome editing rates obtained from ddPCR, plants with relatively high editing rates underwent further analysis through restriction enzyme digestion and PCR amplification. The resulting PCR products, encompassing the specific small, guided RNA target locus, were subcloned and sequenced to confirm successful genome editing. This successful application of the CRISPR/Cas9 system to edit the SPL9 gene within a multiplex genomic context provides valuable insights into the future application of this technology in alfalfa improvement. Nonetheless, the overall editing efficiency observed in the polyploid alfalfa genome was lower compared to that in less complex plant genomes, necessitating further refinement of CRISPR methodologies for enhanced genome editing efficacy in this species.

Wolabu [68] constructed multiplex gRNA-CRISPR/Cas9 vectors employing a polycistronic tRNA-gRNA approach targeting the *Medicago sativa stay-green* (*MsSGR*) gene. They demonstrated that substituting the CaMV35S promoter with the Arabidopsis ubiquitin promoter (*AtUBQ10*) to drive Cas9 expression in the multiplex gRNA system significantly enhanced genome editing efficiency. This optimized multiplex system achieved a genotypic mutagenesis efficiency of 75%, representing a 30-fold increase over single gRNA vectors. Importantly, phenotypic changes were readily observable in the mutants, with a phenotypic mutation efficiency of 68%. This highly efficient multiplex gRNA-CRISPR/Cas9 genome editing system enabled the generation of homozygous mutants with complete knockout of the four allelic copies in the T0 generation, effectively overcoming a major barrier to the utilization of genome editing for alfalfa improvement.

Subsequently, they investigated the potential for delaying flowering in alfalfa through multiplex CRISPR/Cas9-mediated mutagenesis of *FLOWERING LOCUS Ta1* (*MsFTa1*), a key floral integrator and activator gene. Four guide RNAs (gRNAs) were designed and clustered within a polycistronic tRNA-gRNA framework, which was then introduced into alfalfa via Agrobacterium-mediated transformation. Ninety-six putative mutant lines were identified through gene sequencing and characterized for delayed flowering time and associated desirable agronomic traits. Phenotypic assessment under long-day conditions revealed 22 independent mutant lines exhibiting delayed flowering compared to controls. Six independent MsFTa1 lines, harboring mutations in all four alleles of MsFTa1, displayed significantly enhanced forage biomass yields—up to a 78% increase in fresh weight and 76% in dry weight—relative to controls. Additionally, many of these lines exhibited reduced lignin, acid detergent fiber (ADF), and neutral detergent fiber (NDF) content, alongside significantly elevated crude protein (CP) and mineral contents, particularly in stem tissues.

Recently, Wolabu [69] demonstrated that mutating the *COUMARATE 3-HYDROXYLASE* gene in alfalfa using multiplex CRISPR/Cas9 leads to reduced lignin deposition and improved forage quality. These advancements underscore the potential of CRISPR technology for enhancing genetic improvement in alfalfa, facilitating the development of cultivars with superior traits for sustainable agricultural practices.

## 5. Conclusion and Future Perspectives

CRISPR/Cas genome editing presents a transformative approach for enhancing alfalfa breeding by precisely targeting genes involved in nutrient signaling and abiotic stress tolerance. Key genes like *AKT1*, *NRT1.1*, and *ANR1* can be edited to improve nutrient uptake efficiency and root development, optimizing alfalfa’s adaptability to nutrient-deficient environments. Additionally, CRISPR’s ability to fine-tune gene expression through targeted knockouts, such as those in promoter regions of *NRT2.1* and *NRT2.2*, offers a sophisticated method to enhance nitrogen uptake without completely disabling the gene. Furthermore, targeting genes like *CIPK23* and *HRS1* could boost the plant’s resilience to potassium and phosphate deficiencies. Future research should focus on expanding beyond these targets to explore broader gene networks and utilize high-throughput phenotyping for novel gene discovery. As CRISPR techniques evolve, compliance with regulatory standards will be essential to enable the commercialization of CRISPR-modified alfalfa with enhanced stress tolerance, nutrient efficiency, and productivity.

An important consideration in the application of CRISPR/Cas genome editing is the potential risk of gene flow from cultivated alfalfa to its wild relatives. While gene-edited crops generally present a lower risk of unintended traits compared to traditional genetically modified crops, the possibility of gene transfer remains a concern, particularly for traits related to biotic and abiotic stress tolerance. Gene flow could potentially provide unintended advantages to wild populations, leading to ecological imbalances. To mitigate this risk, strategies such as isolation distances, containment measures, and ongoing monitoring are essential to limit the spread of edited traits. Furthermore, regulatory frameworks should be established to ensure responsible gene editing practices in alfalfa, including thorough environmental impact assessments. By adopting these precautionary measures, the benefits of CRISPR/Cas technology can be maximized while minimizing ecological risks.

## Data Availability

No new data were created or analyzed in this study. Data sharing is not applicable to this article.
